# Possibility of Wild Boar Harm Occurring in Five Provinces of Northwest China

**DOI:** 10.3390/ani13243788

**Published:** 2023-12-08

**Authors:** Penghui Liu, Zhicheng Wang, Kang An, Yuchen Tan, Weihong Ji, Junhu Su

**Affiliations:** 1 College of Grassland Science, Gansu Agricultural University, Lanzhou 730070, China; penghui_liu0727@163.com (P.L.); zhicheng_wang0910@163.com (Z.W.); kang_an1997@163.com (K.A.); sasuke421339218@163.com (Y.T.); 2 Key Laboratory of Grassland Ecosystem, Ministry of Education, Gansu Agricultural University, Lanzhou 730070, China; 3 Gansu Agricultural University-Massey University Research Centre for Grassland Biodiversity, Gansu Agricultural University, Lanzhou 730070, China; j.j.weihong@massey.ac.nz

**Keywords:** wild boar, habitat, human–wildlife conflict, species distribution model, superposition analysis

## Abstract

**Simple Summary:**

With the implementation of China’s ecological projects and related policies, the population of wild boars has rapidly increased, leading to an increased possibility of conflicts with humans. Thus, it is crucial to understand the distribution and habitat selection of wild boars in northwest China for the control and management of this species. This study used the maximum entropy model (MaxEnt) to analyze the potential distribution areas of wild boars in the northwest region under the current environmental conditions and compare changes in land use in their habitats over time. The findings revealed that wild boars prefer habitats dominated by cultivated land, woodland, and grassland. Precipitation seasonality, elevation, and human footprint index were identified as the main environmental factors influencing their habitat selection. These findings indicate that wild boar habitat selection results from comprehensive consideration of the environment, providing a theoretical reference for guiding ecological prevention measures and population management strategies for wild boars in the northwestern region.

**Abstract:**

With the implementation of ecological engineering projects and related policies in China, wild boar (*Sus scrofa*) populations have surged, leading to increasingly serious conflicts with humans. We evaluated their potential habitat changes from the perspective of environmental suitability. To elucidate the suitable habitat characteristics for wild boars, we obtained data from 79 sites in five provinces in northwest China using database retrieval, human–wildlife conflict (HWC) incident questionnaires, and document retrieval. Thus, 10 environmental variables with lower correlation were selected, and potentially suitable distribution areas for wild boars under the current climate scenario were predicted based on the maximum entropy model. These areas were superimposed with different land use types in different periods to explore habitat selection. Precipitation seasonality (26.40%), human footprint index (16.50%), and elevation (11.90%) were the main environmental factors affecting wild boar distribution. The areas with high potential suitability for wild boars were mainly in the southeast and northwest of the region (total area of 2.63 × 10^5^ km^2^). The land use types in the high-suitability zones are mainly woodland and grassland with high coverage, canopy density, and cultivated land borders. This study provides a reference for the effective prevention of HWC and management of wild boars.

## 1. Introduction

A habitat is an area inhabited by organisms. It is composed of abiotic elements (e.g., weather, topography, and hydrology) and biotic elements (e.g., vegetation structure and composition, interspecific pressure, competition, race antagonism, prevalence of diseases) that can satisfy the basic requirements of an organism, such that it can complete an entire life history event (e.g., avoiding predators, foraging, mating, and raising young) [1,2]. Habitat selection for individual organisms is crucial, usually requiring them to choose or occupy a set of non-random locations with ambient resources, ideal climatic conditions, and protection from enemies [3], and habitat differences have significant effects on species distribution, density, and survival rates [4]. The potential ability of individual organisms to survive and develop can be understood using habitat suitability assessments [5,6].

Most of northwestern China has arid and semiarid regions with diverse and complex landscapes, many mountainous areas, year-round droughts, and low rainfall. The total land area of the region is 3.04 × 10^6^ km^2^. Forest and grassland areas account for 7.60% and 36.26% of the area, respectively, and unused land (e.g., deserts) accounts for 46.46%. Moreover, the vegetation coverage rate is low, and the ecological environment is fragile [7].

As the human population increases and the scale of industry expands, activities such as deforestation, grassland destruction, and farmland reclamation cause serious damage to the ecological environment. According to statistics from 1970 to 1985, the area of forest resources destroyed in five northwestern provinces (Gansu, Shaanxi, Qinghai, the Ningxia Hui Autonomous Region, and the Xinjiang Uygur Autonomous Region) was as high as 3.10 × 10^4^ km^2^, accounting for 19.75% of the total forest area in the region. As of 1999, the grasslands in these provinces were degraded to different degrees. More than 50% of the grasslands were seriously degraded in Shaanxi Province, where the degradation was the most serious, and the area of degraded grassland was 2.37 × 10^4^ km^2^. The total degraded area in the remaining four provinces, according to incomplete statistics, was 2.77 × 10^5^ km^2^ [7,8].

The destruction of forests and grasslands has forced wildlife to migrate from habitats unsuitable for survival, resulting in an ongoing human–wildlife conflict (HWC) [9]. Favorable conditions for wildlife survival were created by the promulgation of the Environmental Protection Law (for trial implementation) in 1979, the “Gun Control Law” promulgated in 1996, and Grain for Green and Grassland in the agricultural–pastoral ecotone and forest edge areas in 1999. The number of wild animals is rapidly increasing, and their distribution range is expanding accordingly and leading to an increase in HWC (e.g., elephants in Yunnan, and wild boars and Amur tigers in Changbai Mountain, Huncun, and other areas in Jilin Province.), including the destruction of the cash crops of farmers and herders and the endangerment of the personal safety of residents, disrupting local production and livelihoods [10].

The wild boar (*Sus scrofa* Linnaeus, 1758), originally from Asia and Europe, is the world’s most widely distributed large omnivore [11]. Wild boars inhabit a wide range of habitats, ranging from swamps, forest grasslands, and shrubby wetlands to high-elevation alpine grasslands [12]. Their populations, when at reasonable densities, have a moderating effect on ecosystem functioning in their habitats, including their foraging process and daily activities (e.g., disturbing the soil and trampling), improve the physicochemical properties of the soil, and increase the species richness of the area [13,14,15]. However, when the population density of wild boars is too high and interspecific competition increases, their activity range expands; thus, they conduct their activities, foraging further from their nests, and begin to move into the agro-forestry ecotone and forest edge zones and overlap with areas of human activity, resulting in feeding damage to crops, endangerment of the personal safety of farmers and herders, and substantial economic losses [16,17,18]. Furthermore, wild boars and ticks play important roles in the transmission of African swine fever virus. Wild boars act as hosts for African swine fever virus under natural conditions, and ticks are the primary biological vectors of the virus, who transmit it via their bites. When large-scale farms or self-constructed pigsties located in residential areas overlap with zones containing wild boars, the likelihood of swine fever outbreaks in the vicinity increases [19]. The growth of wild boar populations increases the risk that countries that have eliminated swine fever will experience outbreaks again [20]. Therefore, it is crucial to assess the suitability of wild boar habitats in northwest China, comprehend their quality and environmental aspects, examine the influence of each factor on the distribution of wild boars, and establish a theoretical foundation for HWC assessment, the management of wild boar, the monitoring of wildlife epidemics, and risk appraisal.

Species distribution models are widely used to predict and assess plant and animal habitats and can be divided into two categories based on the evaluation method [21]: the threshold correlation method and the threshold-independent method. Threshold-independent methods, such as maximum entropy models (MaxEnt) and generalized linear models, are more suitable for species distribution evaluation. The MaxEnt model can assess the habitat environment with only a small number of species “occurrence points” and is less sensitive to the covariance of environmental variables. Furthermore, this model is easy to operate and results in a high prediction accuracy.

Wild boars can readily adapt to habitats according to long-term evolution, but differences in habitat constraints (climatic factors, geographical factors, food resources, and human disturbances) have led to differences in the habitats selected by wild boars in different regions. Studies have shown that precipitation, temperature, and human disturbances are decisive factors that influence wild boar habitat selection [22,23,24]. Changes in temperature affect population contraction and dispersal. Suitable temperature conditions, abundant food resources, and a quiet habitat determine the choice of wild boar habitat [25,26]. Wild boars prefer foraging in forested farmland, which provides sufficient food and a high degree of seclusion [27]. Most studies evaluating the suitability of habitats in China for wild boars have been biased toward nature reserves or the southern regions, and, to our knowledge [10,28], few relevant studies are based in the northwestern region. Evaluating potential habitat changes from the perspective of environmental suitability is crucial for reducing HWC and optimizing management strategies.

By evaluating the suitability of wildlife habitats, understanding the survival and distribution of a species is possible, providing a theoretical basis for the protection and management of species populations and their habitats. In this study, we evaluated the habitat suitability of wild boars in the five northwestern provinces of China by collecting data on their natural distribution points; combining environmental, topographic, human disturbance, and food resource data under the current climate scenario; and overlaying the high-suitability areas for wild boars with the land use types of different periods using the ArcGIS (ESRI, Redlands, CA, USA, https://desktop.arcgis.com, accessed on 2 August 2022, v10.8) software. This study aimed to (1) identify the potential geographic distribution of wild boars in northwestern China and the factors affecting it; (2) determine areas that provide highly suitable conditions for wild boars and the main land use types in different periods; and (3) propose future wild boar management strategies based on the suitability evaluation. This study provides a reference for researchers and policymakers on wild boar population management, the prevention of HWC, and public health and safety.

## 2. Materials and Methods

### 2.1. Acquisition and Processing of Geographic Distribution Data

**Figure 1:**
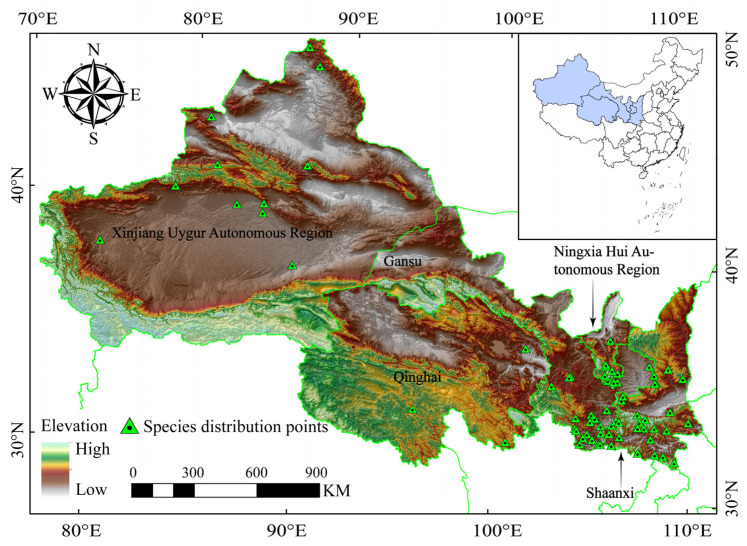
Records of presence of wild boars in five northwestern provinces.

The data on the distribution sites of wild boars used in this study were collected from the following three sources: (1) China National Specimen Resource Platform (http://www.nsii.org.cn, accessed on 13 May 2023) and National Animal Specimen Resource Library (http://museum.ioz.ac.cn, accessed on 13 May 2023); (2) the research team’s recorded GPS locations of crop damage incidents caused by wild boars and human–wild boar encounters during a wildlife incident survey conducted from June to July 2022; and (3) the China National Knowledge Infrastructure database (CNKI, https://www.cnki.net, accessed on 25 May 2023) [29,30,31]. To avoid overfitting the model due to points with closer spatial distances, we excluded duplicate and adjacent GPS positions [32], ultimately retaining 79 GPS positions in the .csv format (Table S1). Taking the entire region of the five northwestern provinces of China as the background set, combined with the distribution points of the aforementioned sources, the distribution pattern of wild boars in the five northwestern provinces was determined (Figure 1).

### 2.2. Environmental Variables

We selected 24 environmental factors from four categories, terrain (elevation, slope, and aspect), climate (19 bioclimatic variables), vegetation (normalized difference vegetation index [NDVI]), and human interference (human footprint index), as influencing factors for the wild boar habitat suitability evaluation. The topographic and climate factors were derived from the Worldclim database (http://www.worldclim.org, accessed on 10 May 2023) [33], and included temperature and precipitation. The terrain factors were obtained using ArcGIS v10.8, and using elevation layers. The HFI data were sourced from the Global Human Footprint Dataset (http://sedac.ciesin.columbia.edu/data, accessed on 11 May 2023) produced by the Wildlife Conservation Society and the Center for International Earth Science Information Network of Columbia University in 2016, and were derived from the impact of human activities such as land use, population density, infrastructure construction, such as roads and railways, and farmland grazing [34]. The vegetation data and land use types were obtained from the Resources, Environment, and Data Center of the Chinese Academy of Sciences (https://www.resdc.cn, accessed on 10 July 2022). The spatial distribution dataset on China’s annual vegetation index was processed to obtain the NDVI from 1980 to 2018. The land use types were derived from the China Land Use Remote Sensing Monitoring Dataset, using a two-level classification system. The first level was divided into six categories: cultivated land, forested land, grassland, water area, construction land, and unused land. The second level was further divided into 25 types based on the first level (Table S2) [35]. Three phases of land use remote sensing data from the five northwestern provinces in 1980, 1990, and 2000 were selected for analysis. All data were resampled into a unified coordinate system and resolution and then converted into ASC format for subsequent analysis. Environmental variables were eliminated using the SPSS software (IBM, New York, NY, USA, https://www.ibm.com/cn-zh/spss/, accessed on 16 July 2023, R26.0.0.0). Next, we removed the 14 variables using a Pearson’s correlation coefficient |r| > 0.80 for subsequent modeling (Tables S3 and S4); then, 10 environmental variables were selected to construct the boar ecological niche model (Table 1).

### 2.3. Maximum Entropy Modeling and Model Evaluation

The collected longitude and latitude data on the wild boar distribution points were imported into the MaxEnt v3.3.4 model in .csv format, and the “ENMelval” package in R was used to optimize the feature combination parameters and regularization multiplier [36,37]. Six feature combinations (L, LQ, H, LQH, LQHP, and LQHPT; L = linear, Q = quadratic, H = hinge, P = product, and T = threshold) and regularization multipliers (0.5, 1.0, 1.5, 2.0, 2.5, 3.0, 3.5, and 4.0) were used to adjust the model to optimize its prediction ability [38]. We used 75% of the distribution points as the training set and 25% as the test set [39]. We selected jackknife resampling, LQHPT (Table S5), response curves, and prediction images generated using the prediction parameter options for the analyses. The number of model iterations was 5000, the regularization multiplier was 3.0, and the number of repetitions was 10. The other parameters were set to their original default values. Based on the receiver operating characteristic curve of the model, its performance was evaluated by calculating the area under the curve and continuous Boyce index.

### 2.4. Classification of Suitable Living Areas and Their Overlap with Land Use Types

We classified the ecological suitability of habitats for wild boars into four levels using the ArcGIS 10.8 software) according to the natural break classification method [40]: non-suitable areas, low-suitability areas, medium-suitability areas, and high-suitability areas. Using the conversion tool of ArcGIS 10.8, the ASCII-encoded file that copied the average prediction results of 10 models was converted into a grid format to obtain a distribution map of potentially suitable areas for wild boars. A grid calculator was used to calculate the area and proportion of suitable areas at each level. The land use types were determined using overlay analysis of high-suitability areas during three periods: 1980, 1990, and 2000.

## 3. Results

### 3.1. Accuracy Analysis of Maxent Model

The accuracy of the prediction results of the MaxEnt model was verified using receiver operating characteristic curve analysis. The area under curve (AUC) of the wild boar habitat prediction model in the five northwestern provinces was 0.954, and the continuous Boyce index (CBI) was 0.923. The model results showed excellent performance, fulfilled the accuracy requirements, and had relatively good stability after 10 replications (Table S5).

### 3.2. Analysis of the Contribution Rate of Environmental Variables

**Figure 2:**
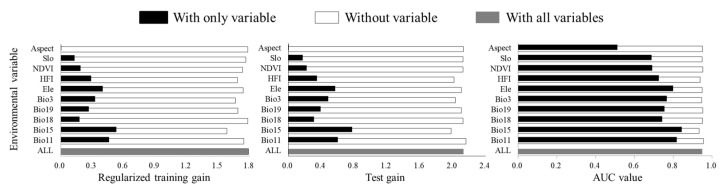
Importance of environmental variables evaluated using jackknife resampling.

Analyzing the environmental variable contribution rate showed that Bio15, HFI, and Elev had the highest contribution rates (26.40%, 16.50%, and 11.90%, respectively), indicating that they were the main factors affecting the distribution of wild boars in the northwest region. Bio3, Bio19, and NDVI were secondary factors, with contribution rates of 11.50%, 9.60%, and 9.40%. The contribution rates of Bio18, Bio11, Slo, and Asp were relatively low, with a cumulative contribution rate of 14.70%, indicating that these six variables have a relatively small impact on the suitability of habitats for wild boars (Table 2). According to the results of jackknife resampling, under the current climate scenario, Bio11, Bio15, and Elev substantially affected the distribution prediction results, followed by HFI, Bio3, Bio19, and NDVI, and Slo, Bio18, and Asp had a relatively small impact on the distribution prediction results (Figure 2).

**Figure 3:**
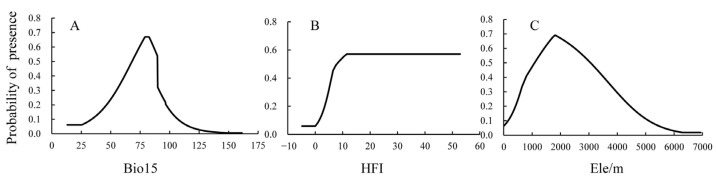
Response curves for major environmental variables. (**A**) Bio15: precipitation seasonality; (**B**) HFI: human footprint index; (**C**) Ele: elevation.

We plotted the response curve of the main environmental factors in the model and found that the probability of wild boar existence first increased and then decreased with increasing Bio15 and Elev. The probability of HFI existence remained unchanged after reaching a certain level, indicating a certain correlation between the influences of HFI, Bio5, and Elev on the probability of wild boar existence. When 68.08 < Bio15 < 89.57 and 1085 m < Elev < 3054 m, the probability of wild boar existence is >50%, and when HFI > 12.60, the probability of wild boar existence is constant (Figure 3).

### 3.3. Potential Suitable Habitat Distribution

**Figure 4:**
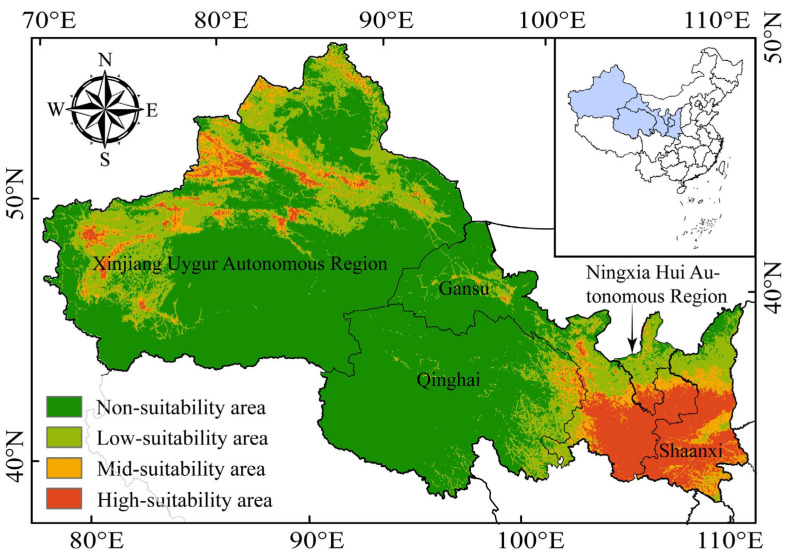
Potential distribution of wild boars in northwest China.

The prediction results of the MaxEnt model indicated that the highly suitable areas are mainly distributed in Qingyang in the southern and eastern of Gansu Province, the southern part of the Ningxia Hui Autonomous Region, and the Qinling Nature Reserve south of Yan’An City in Shaanxi Province as the center. The western edge zone was a main distribution area, as was the intersection zone between the Tianshan Mountains and the northern edge of the Xinjiang region. The remaining were scattered in the northwestern edge zone centered on the Mu Tarim Basin in the Xinjiang Uygur Autonomous Region, the Altay Mountains in Altay, a small part of Guoluo, Hainan, and Haixi Tibetan Autonomous Prefecture in Qinghai Province, Wuwei City in Gansu Province, and Hami and Tongmenguan in the Xinjiang Uygur Autonomous Region (Figure 4).

The high-suitability areas covered 2.63 × 10^5^ km^2^, accounting for 8.54% of the total area of the five northwestern provinces, and were mainly in Gansu Province, Shaanxi Province, the Xinjiang Uygur Autonomous Region, and the Ningxia Hui Autonomous Region. The proportion of medium-suitability areas was 6.98%, and that of the low-suitability areas was 6.73 × 10^5^ km^2^. The non-suitable areas were mainly located in the northern regions of Qinghai Province, the Xinjiang Uygur Autonomous Region, and Gansu Province, accounting for 62.64% of the total area of the five provinces, with an area of up to 1.93 × 10^6^ km^2^ (Table 3).

### 3.4. Overlapping Analysis of High-Suitability Areas and Land Use Types

The results showed that the main habitat types of high-suitability areas were cultivated land, forest land, and grassland. Grasslands were the preferred habitat. The grassland areas in 1980, 1990, and 2000 accounted for 40.99%, 41.27%, and 40.06% of the total high-suitability areas, respectively, with an average preferred habitat area of 1.07 × 10^5^ km^2^. Our model results indicated that wild boars also have high selectivity toward arable land and forest land. From 1980 to 2000, the cultivated land area in high-suitability areas increased to 8.64 × 10^4^ km^2^, accounting for an increase of 162 km^2^; however, there was no significant change in woodland area (Table 4).

**Figure 5:**
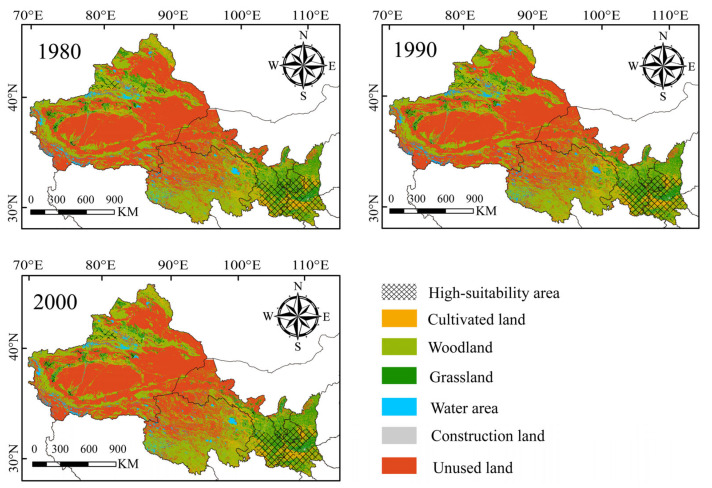
Land use types in different periods and high-suitability areas for wild boars under the current climate (black grid area).

By calculating the secondary classification area of land use types in different periods, we found that the preferred habitat for wild boars was medium-coverage grassland with coverage greater than 20–50%, followed by cultivated land with dry land as the main habitat, and forested land with a canopy closure >30%. In addition, the area of medium-coverage grassland in the medium- to high-suitability areas has a decreasing trend for the past 30 years, from 5.45 × 10^4^ km^2^ in 1980 to 5.40 × 10^4^ km^2^ in 2000. The change in forested land area was not significant, and the trend in dry land area change was the opposite of that of the previous two. The area of dry land has increased by 1696 km^2^ over the past 30 years (Table 5, Figure 5).

## 4. Discussion

### 4.1. Environmental Variables Affecting the Distribution of Wild Boars

The area under curve (AUC) of our prediction model was 0.958, and the continuous Boyce index (CBI) was 0.923, indicating good prediction results. Precipitation seasonality, human footprint index, and elevation were important factors affecting the habitat suitability of wild boars in the five northwestern provinces. The high-suitability areas predicted by the model had the characteristics of moderate altitude, abundant precipitation, and a certain degree of human activity. Generally, wild animals tend to avoid areas frequented by humans to reduce human interference. But the favorable environment around human settlements can provide them with sufficient water and food resources. In addition, fewer predators and competitors around human settlements also reduce the risk of predation for and energy consumption of wild boars. The analysis indicates that the likelihood of wild boars appearing in regions with a low human footprint index is not high, which aligns with our expectations. This result does not indicate that wild boars are absent in areas unexplored by humans. A possibility is that the lives of wild boars in areas that fulfill the aforementioned climatic and terrain conditions have not been disturbed by humans. Most wild boars live between grasslands and mountain forests, and a few areas of wild boar activity on the edges of forests and grasslands overlap with areas of human activity. In summer and autumn, food is more abundant on farmland, and the surrounding forests, shrublands, and other areas have a high canopy density, which can provide shelter for wild boars. Under such conditions, boars will venture into farmland areas to forage, during which the likelihood of boars encountering humans increases. Studies have shown that conflicts between humans and wild boars occur in certain regions, and the risk of harm caused by wild boars to farmland at the edges of agricultural and forestry areas is relatively high [41,42]. Therefore, prevention and control measures being implemented by farmers and herders to protect crops from being eaten or destroyed may have an indirect impact on the ecological niche of wild boars, reducing the probability of human–boar conflicts.

Precipitation seasonality plays a decisive role in the composition and productivity of plant communities [43]. Plants are one of the main sources of food for wild boars and a decisive factor in their habitat selection. Using long-term monitoring and research, Li et al. [44] found that precipitation seasonality is one of the key factors determining the distribution of vertebrates. The vertical climate characteristics caused by lower elevation are more obvious, and water and heat conditions are redistributed with changes in elevation. Consequently, there is a close relationship between elevation and the vertical distribution of zonal vegetation. Additionally, there are seasonal differences in plant resource gradients, leading to wild boars choosing different elevations in different seasons [45]. Furthermore, relatively high elevations tend to have less human interference. Therefore, the appropriate elevation is selected by wild boars based on comprehensive consideration of food and living environments.

### 4.2. Current Potential Distribution

The land use types of high-suitability areas for wild boars in northwestern China were mainly grassland, cultivated land, and forest land, with abundant food resources, less human interference, and a high canopy density. The grassland types were mainly medium- to high-coverage grasslands, which are the main foraging grounds for wild boars. Abundant plant resources provide favorable conditions for the survival and reproduction of wild boars. The impact that wild boars have on grasslands is multifaceted. Wild boars churning the soil during foraging can promote the growth of grassland vegetation roots and nutrient cycling [46]. However, in agricultural–pastoral ecotones, wild boar activity causes substantial crop damage and indirect losses [47]. The results showed that the grassland area in high-suitability areas is decreasing annually, and further research is necessary to determine whether the rapid growth of wild boar populations and habitat expansion pose potential risks to China’s grassland ecosystems. The type of cultivated land is mainly dry land. Owing to the diverse feeding habits of wild boars, various crops are within their diet range, and the food in farmland is abundant and easy to eat. Wild boars feeding on crops can exacerbate conflicts between humans and wild boars. The cultivated land in the forest edge area is most severely damaged by wild boars, and some farmers and herdsmen will use hunting or even poisoning to prevent the destruction of farmland [48,49,50]. In multiple investigations of wild boar accidents, over 80% of farmers and herdsmen stated that the number of wild boars has increased annually, indicating that the activity areas of wild boars are also expanding [49,51,52]. The cultivated land area in the potential high-suitability areas for wild boars in the northwestern region has increased annually, increasing by nearly 300 km^2^ from 1980 to 2000. The proportion of grassland and forestland is decreasing annually, and the area of cultivated land is increasing annually (1980–2000), However, with the implementation of the project of Grain for Green and Grassland, the restoration of the original habitat of wild boars (woodland and grassland) will contribute to the growth of the wild boar population (Table S6), with an increased likelihood of human encounters with wild boars.

Possibly due to the distinct regional characteristics of the collected species distribution points in the study area, the output results of the migration trend of wild boars in the northwestern region generated by our model might not have been ideal, preventing the accurate determination of the migration trend of this species. Information on other factors, such as railways, highways, and rivers, would provide more robust theoretical support for predicting the potential distribution areas of wild boars, but considering the impact of wild boars on humans, the HFI will more intuitively demonstrate the impact of wild boars on humans. Additionally, owing to data limitations, high-suitability areas were observed in aquatic environments; however, the intersection between land and water might be suitable for the survival of wild boars, with the availability of water emerging as a key factor influencing wild boar distribution. Therefore, the results of this study require detailed data support and long-term monitoring.

### 4.3. Management Suggestions and Measures for Future Wild Boar Populations

Crop destruction by wild boars has been identified as a major cause of HWC in many countries around the world. Based on the environmental factors affecting the distribution of wild boars and the land use in potential high-suitability areas, our findings indicated an elevated probability of wild boar presence in forested areas, grasslands, and cultivated land proximal to areas with frequent human activities (Figure S1), especially in areas with available water sources. Such areas should take measures to avoid HWC being caused by wild boars. It has been shown that many farmers in agro-forestry areas have adopted non-lethal measures and set up “rapid response teams” for patrolling, proving effective in mitigating the crop damage attributable to wild boars [41,53]. These teams employed gongs and loudspeakers to deter, disturb, and guide wild boars foraging near farmland away from the area. For arable land and ecological restoration areas in high-suitability zones that are frequently damaged by wild boars, wild boars can be prevented from entering by increasing the number of patrols with hounds, splashing pig manure, clearing the surrounding dense vegetation and building physical barriers (e.g., electrified fences, digging trenches), or installing security-controlled intimidation devices [54,55]. Considering that the hindrance and deterrence of the above measures on wild boars will diminish over time, a system of wild boar population monitoring and incident counting should be implemented to increase the insights into the development of wild boar populations, which will facilitate the implementation of controlled hunting via management using a planned approach to control wild boar populations and reduce wild-boar-induced HWC. It is also recommended to implement an eco-compensation program and an insurance scheme to compensate for the economic losses caused by wild boars. Woodlands and grasslands serve as the original habitat for wild boars. A relatively moderate approach should be used to manage wild boars from the perspective of population ecology in its low-suitability areas and medium-suitability areas. Infrared camera technology and eDNA technology [56] can be used to conduct more in-depth surveys of wild boars to achieve long-term monitoring and management of wild boar species distribution. In this process, traditional awareness building such as Social and Behavioral Change Campaigns (SBCCs) can be conducted in the periphery of towns and village in the forest steppe to increase residents’ knowledge of wildlife conservation regulations and their tolerance of wild boars, by combining the above measures, developing a manual on the prevention of wildlife damage, and using scientific and effective means to protect the personal safety of the population and agricultural property.

## 5. Conclusions

This study found that the human footprint index, seasonal precipitation, and elevation were the main environmental factors affecting the distribution of wild boars in five provinces of northwestern China. The high-suitability areas for wild boars were mainly distributed in the east and northwest, accounting for 5.7% of the area. The high-suitability areas were mainly composed of forest and grassland with high coverage and canopy closure, as well as adjacent farmland. Notably, the area of forest and grassland is decreasing annually, and the area of farmland is increasing annually; thus, we found that the possibility of harm to wild boars in high-suitability areas was relatively high. Assessment of the potential habitat changes from the perspective of environmental suitability and the prediction of potential habitats highlighted important environmental factors and potential distribution areas that determine the distribution of wild boars. Finally, we proposed targeted measures to reduce conflicts between humans and boars and effectively manage wild boar populations, providing a theoretical reference for the ecological prevention and population management of wild boars in the northwestern region.

## Figures and Tables

**Table 1:** Environmental variables for modeling wild boar habitat suitability.

Environment Variable	Abbreviation	Unit
Isothermality	Bio3	—
Mean temperature of coldest quarter	Bio11	°C
Precipitation seasonality	Bio15	mm
Precipitation of warmest quarter	Bio18	mm
Precipitation of coldest quarter	Bio19	mm
Human footprint index	HFI	—
Elevation	Elev	m
Slope	Slo	°
Aspect	Asp	—
Normalized difference vegetation index	NDVI	—

**Table 2:** Contribution rates of environmental factors.

Environment Variable	Percent Contribution
Bio15	26.40
HFI	16.50
Elev	11.90
Bio3	11.50
Bio19	9.60
NDVI	9.40
Bio18	8.00
Bio11	4.80
Slo	1.60
Asp	0.30

Note: Bio11: mean temperature of coldest quarter, Bio15: precipitation seasonality, Bio18: precipitation of warmest quarter, Bio19: precipitation of coldest quarter, HFI: human footprint index, Elev: elevation, Slo: slope, Asp: aspect, NDVI: normalized difference vegetation index.

**Table 3:** Area and proportion of suitable habitats. HSA high-suitability areas, MSA: medium-suitability areas, LSA: low-suitability areas, NSA: non-suitable areas.

Suitable Grade	Dimension/km^2^	Percentage/%
HSA	2.63 × 10^5^	8.54
MSA	2.15 × 10^5^	6.98
LSA	6.73 × 10^5^	21.84
NSA	1.93 × 10^6^	62.64

**Table 4:** Proportion of area of first-class land use types in potential high-suitability areas during different periods. (Unit: ×10^5^ km^2^).

	Cultivated Land	Woodland	Grassland	Water Area	Construction Land	Unused Land
1980	0.8475	0.6163	1.0719	0.0178	0.0427	0.0190
1990	0.8505	0.6055	1.0808	0.0164	0.0392	0.0262
2000	0.8637	0.6166	1.0476	0.0176	0.0469	0.0226

**Table 5:** Areas of secondary land use types in potentially high-suitability areas in different periods. (Unit: ×10^4^ km^2^).

Land Use Type		1980	1990	2000
Cultivated land	Paddy field	0.6203	0.6391	0.6134
	Dry land	7.8544	7.8664	8.0240
Woodland	Forested land	2.9402	2.8666	2.9351
	Shrubbery	1.8676	1.8676	1.8762
	Open woodland	1.3107	1.2765	1.3813
	Other forest land	0.0377	0.0445	0.0445
Grassland	High-coverage grassland	3.6873	3.7918	3.6068
	Medium-coverage grassland	5.4487	5.4007	5.3956
	Low coverage grassland	1.5832	1.6157	1.4735
Water area	Water area	0.1782	0.1645	0.1765
Construction land	Urban land	0.0633	0.0805	0.0719
	Rural residential area	0.3564	0.3050	0.3907
	Other construction land	0.0069	0.0069	0.0069
Unused land	Unused land	0.1902	0.2621	0.2261

## Data Availability

The data presented in article and supplementary material are available on request from the corresponding author.

## Supplementary Materials

The following supporting information can be downloaded at: https://www.mdpi.com/article/10.3390/ani13243788/s1, Figure S1: Human Footprint Index of Northwest China; Table S1: Wild boar occurrence coordinates on the northwest China; Table S2: Land use type classification system. Table S3: Environmental factors used in the present study. Tabke S4. Correlation of environment variaBles. Table S5. The “ENMeval” package runs the optimal parameter results of the MaxEnt model. Table S6. Areas of secondary land-use types in potentially high-suitability areas in 2015.
