# Supplementary figures and images for: Possibility of Wild Boar Harm Occurring in Five Provinces of Northwest China
Source: Animals (Basel). 2023 Dec 8;13(24):3788. doi: 10.3390/ani13243788 (PMC10741053; doi:10.3390/ani13243788)

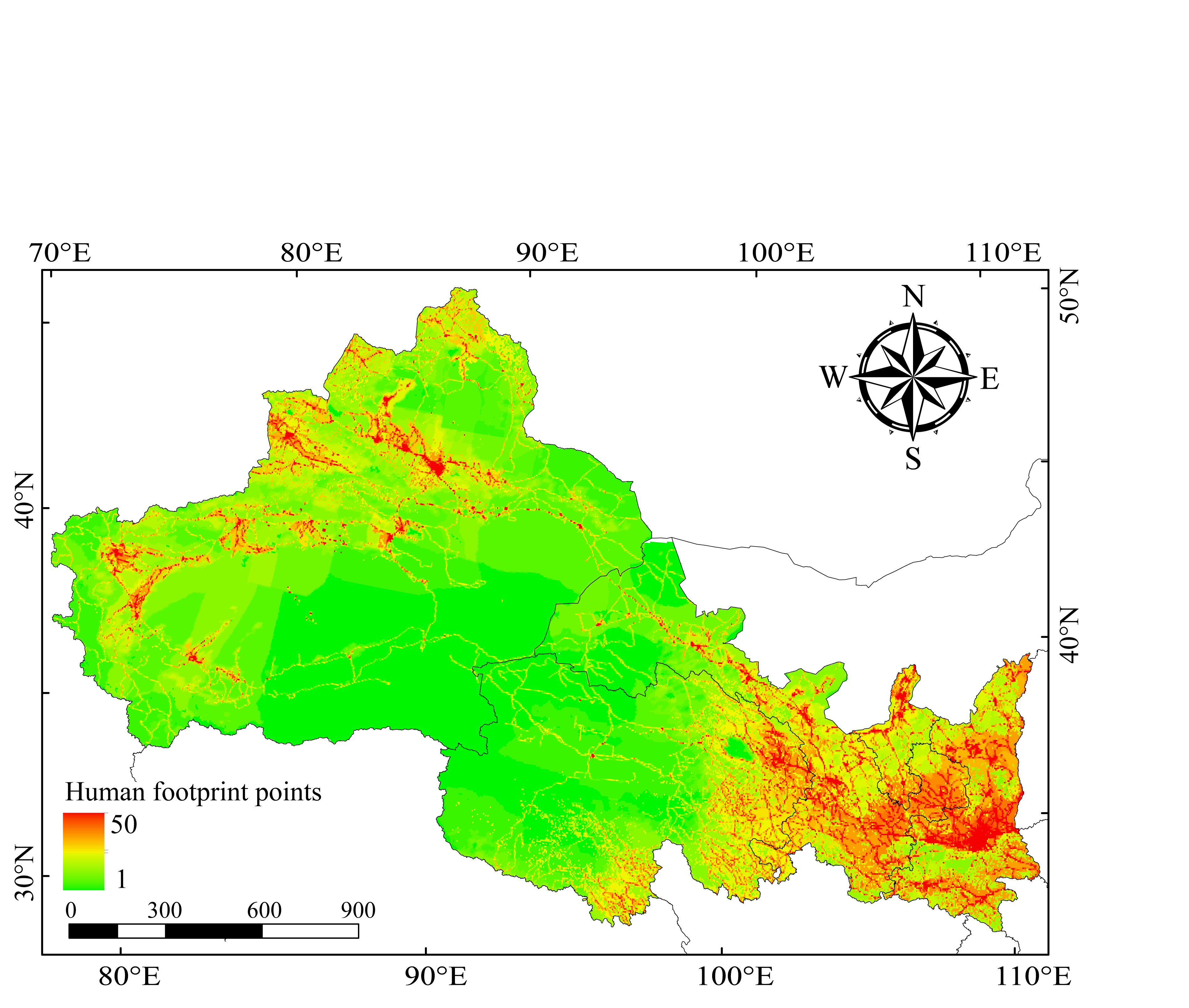

Supplement: Supplementary file 1 [file animals-13-03788-s001.zip › Figure S1.jpg]
